# Protein–protein interaction site prediction by model ensembling with hybrid feature and self-attention

**DOI:** 10.1186/s12859-023-05592-7

**Published:** 2023-12-05

**Authors:** Hanhan Cong, Hong Liu, Yi Cao, Cheng Liang, Yuehui Chen

**Affiliations:** 1 School of Information Science and Engineering, Shandong Normal University, Jinan, China; 2 Shandong Provincial Key Laboratory for Novel Distributed Computer Software Technology, Jinan, China; 3 School of Information Science and Engineering, University of Jinan, Jinan, China; 4 Shandong Provincial Key Laboratory of Network Based Intelligent Computing, Jinan, China

**Keywords:** Protein–protein interaction, Hybrid feature, Self-attention, Integration framework

## Abstract

**Background:**

Protein–protein interactions (PPIs) are crucial in various biological functions and cellular processes. Thus, many computational approaches have been proposed to predict PPI sites. Although significant progress has been made, these methods still have limitations in encoding the characteristics of each amino acid in sequences. Many feature extraction methods rely on the sliding window technique, which simply merges all the features of residues into a vector. The importance of some key residues may be weakened in the feature vector, leading to poor performance.

**Results:**

We propose a novel sequence-based method for PPI sites prediction. The new network model, PPINet, contains multiple feature processing paths. For a residue, the PPINet extracts the features of the targeted residue and its context separately. These two types of features are processed by two paths in the network and combined to form a protein representation, where the two types of features are of relatively equal importance. The model ensembling technique is applied to make use of more features. The base models are trained with different features and then ensembled via stacking. In addition, a data balancing strategy is presented, by which our model can get significant improvement on highly unbalanced data.

**Conclusion:**

The proposed method is evaluated on a fused dataset constructed from Dset186, Dset_72, and PDBset_164, as well as the public Dset_448 dataset. Compared with current state-of-the-art methods, the performance of our method is better than the others. In the most important metrics, such as AUPRC and recall, it surpasses the second-best programmer on the latter dataset by 6.9% and 4.7%, respectively. We also demonstrated that the improvement is essentially due to using the ensemble model, especially, the hybrid feature. We share our code for reproducibility and future research at https://github.com/CandiceCong/StackingPPINet.

## Background

Protein–protein interactions (PPIs) play a crucial role in various biological functions and cellular processes [1], such as signal transduction, immunological recognition, metabolism [2] etc. During PPIs, some interfaces are formed at particular protein residues, called protein–protein interaction sites [3]. Therefore, identifying those sites are essential to reveal the key mechanisms of PPIs and beneficial to modern drug design [4, 5]. However, via experiments, PPI sites identification requires high-end devices and accurate manipulations, being time-consuming and expensive. As an economic and efficient alternative, computational methods [6] have been widely applied. In particular, data-driven methods can provide competitive results by leveraging machine learning and modern deep learning techniques [7–10]. Existing computational approaches can be roughly divided into partner-independent prediction [11] and partner-specific prediction [12]. In addition, according to the feature information, partner-independent prediction can be further divided into structure-based methods [13] and sequence-based methods [14]. Structure-based methods usually need structural details [15]**,** while the structural information for many proteins is currently unavailable in the dataset. With the rapid development of high-throughput sequencing techniques, a growing number of protein sequences can be obtained, which attracts more attention for sequence-based methods [16].

Since the functions of the residues are determined by its physiochemical properties and context [17–19], residues are usually represented by these properties, e.g., accessible surface area [20], protein sequence composition, hydrophilic and hydrophobic index [21]. In addition, evolutionary information [22] and secondary structure information [23] are often incorporated as supplements. To model the local context, sliding window-based methods [24] are widely applied. However, the features of the residues in the window are typically treated equally, which is obviously inaccurate and harms the precise PPI site prediction [25]. Hitherto, many machine learning methods have been proposed to deal with this prediction task, including neural networks (NNs) [26], support vector machines (SVMs) [27], random forests (RF) [28], etc. ISIS [29] is a neural network predictor, which is trained on sequences profiles and structural features predicted from the sequences. SPPIDER [30] employs an SVM, neural network and linear discriminant analysis based on 19 selected features from the sequences. SPRINGS [31] uses mean cumulative hydrophobicity, relative solvent accessibility, and structural features to represent the targeted residue site, and the algorithm uses neural networks for classifier construction. DeepPPISP [32] is an end-to-end deep learning framework that combines local contextual and global sequence features to fulfill the prediction task. Although considerable progress has been achieved, the predictive performance of these methods still needs to be improved [33].

As a matter of fact, most residues in proteins are not PPI sites and thus making the data highly imbalanced [34]. The cascade random forests algorithm (CRF) [35] is first proposed to deal with the problem. It connects multiple random forests in a cascade-like manner, each of which is trained with a balanced training subset that includes all minority samples and a subset of majority samples. However, sampling of training data based on residues level might destroy the completeness of a sequence. SSWRF [36] combines an ensemble of SVMs and sample-weighted random forests to solve the imbalance issue and achieves competing performance. SLSTM utilizes a simplified long short-term memory [37] network to improve the precision of the imbalanced PPI sites. It builds a set of protein sequences, instead of single residues, to retain the entire sequential completeness of each protein. The balancing methods either increase the samples of the minority class or reduces the samples of the majority class, which partly change the data distribution.

In this paper, we proposed a novel sequence-based method for PPI sites prediction. The new network model, namely, PPINet, contains multiple feature processing paths. For a residue, the PPINet extracts the features of the targeted residue and its context separately. These two types of features are processed by two paths and combined to form a protein representation, where the two types of features are of relatively equal importance. The individual PPINets are further ensembled via stacking, by which multiple types of features can be merged. To get high quality hybrid features, the dimensions of the 2 types of the features are adjusted to be equal. Therefore, the bias caused by feature dimensionality can be eliminated during feature fusion. Moreover, a novel data balancing strategy is presented. The majority class of samples (non-interaction sites) are divided into multiple sub-datasets. Each sub-dataset is merged with the entire minority class (interaction sites) to form a balanced dataset, which is used for training. In this way, the consistency of data distributions can be maintained.

Based on the above novelty, the contributions of this paper are as follows:

1. A hybrid feature representation method is proposed to avoid the drawbacks of the traditional sliding window-based methods. The single targeted residue feature and the context feature based sliding window are extracted. They are processed by 2 paths in the PPINet and combined to form a hybrid feature of a protein. This idea is also extended via stacking, where multiple types of features are merged to form a full representation of a protein.
2. A new feature fusion method is proposed, where the feature importance is balanced. In each PPINet, 2 feature vectors are concatenated to form a hybrid feature of a protein. Before concatenation, the dimensions of them are adjusted to be equal so that they can be exploited equally by the model. Therefore, the bias caused by feature dimensionality can be eliminated.

## Methods

**Fig. 1:**
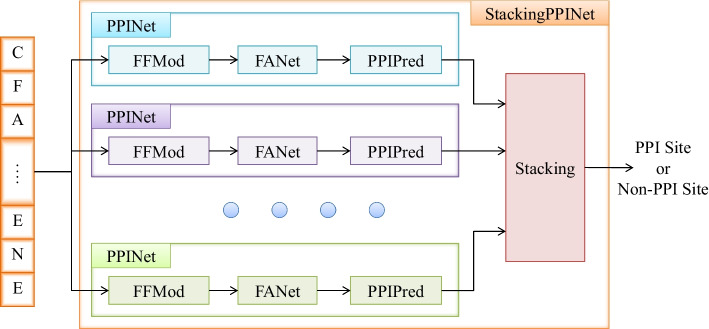
The architecture of StackingPPINet. StackingPPINet contains multiple base classifiers, named PPINets. Each PPINet consists of a feature forming module (FFMod), a feature aggregation network (FANet) and a prediction module (PPIPred). Multiple PPINets are trained independently and ensembled via Stacking

This section describes our proposed ensemble framework (StackingPPINet) for PPI prediction. The architecture of the proposed StackingPPINet is shown in Fig. 1, which fundamentally consists of a group of base classifiers, named PPINets, and a stacking module for ensembling. A PPINet is an independent classifier which predicts whether the targeted amino acid in an input sequence segment is a PPI site. It further contains a feature forming module (FFMod), a feature aggregation network (FANet) and a predictor (PPIPred). The FFMod extracts various low-level features from the input sequence by traditional feature extraction methods. The extracted low-level features are then aggregated into a highly abstracted feature vector with fixed dimension by a FANet. Based on the aggregated feature, decisions are made by the predictor, which is a deep neural network with a binary output. In StackingPPINet architecture, multiple PPINets are first trained independently and then ensembled to enhance the performance and robustness.

### The base classifier for protein–protein interaction site prediction

**Fig. 2:**
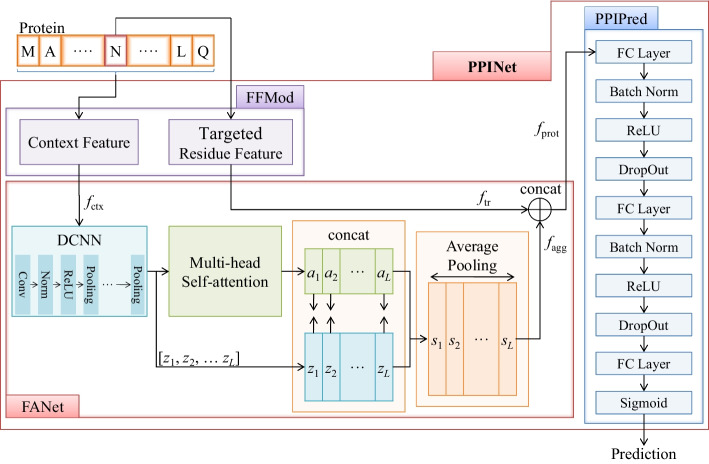
The architecture of PPINet. The feature forming module (FFMod) extracts low level features from the input protein sequence. As an example, K-PseAA and PhyChem are applied in this showcase to extract the targeted residue feature and the context feature respectively. These features are then processed by a feature aggregation network (FANet). Based on the aggregated features, the predictor (PPIPred) performs classification

The base classifiers in the StackingPPINet are PPINets, whose architecture is illustrated in Fig. 2. The FFMod extracts various low-level features from the input protein sequence by traditional feature extraction methods. Specifically, ${f}_{\text{tr}}$ is the targeted residue feature for the target residue, while ${f}_{\text{ctx}}$ is the context feature, which is a series of feature vectors extracted by sliding window-based method. FANet is responsible for aggregating the context feature ${f}_{\text{ctx}}$ into a vector ${f}_{\text{agg}}$ and generating a full protein segment representation, namely ${f}_{\text{prot}}$, by concatenating ${f}_{\text{tr}}$ and ${f}_{\text{agg}}$. Finally, predictions are made by PPINet, which is a deep neural network. Details of those modules are demonstrated in the following subsections.

### The feature forming module

In many existing works, the entire input sequence is converted into a fixed-dimensional feature vector, or is converted into a vector sequence, where the features are extracted separately from each residue. The features of the predicting residue are treated equally to contextual residues. When the context is extended for including more information, the importance of the targeted residue will be weakened, unintendedly harming the model performance.

To address this issue, FFMod extracts the targeted residue feature ${f}_{\text{tr}}$ and the context feature ${f}_{\text{ctx}}$ separately for a residue. For the targeted residue features, FFMod firstly extracts single features and combine them. In this paper, we use six single features, they are one-hot vector [38], position-specific scoring matrix (PSSM) [39], entropy density (Den) [40], physicochemical properties (PhyChem) [41], hydrophilicity and hydrophobicity index (HyIn) [42], and the pseudo amino acid [43] based on K-nearest neighbors (K-PseAA). And the single features are then concatenated in pairs to form combination features for the targeted residue. Finally, there are three combination features for a targeted residue. Table 1 shows the details of these features. For the context feature, it is a connection of multiple residue features in the sliding window. Since it bases sliding window, zero padding is applied for the residues at the ends of the sequence. Although sliding window-based methods can model regional features to some extent, their feature aggregations are restricted by the window size and their simple aggregation patterns. Thus, FFMod only produces low-level features of the input protein sequence, which are not sufficient for PPI sites prediction. The obtained context feature ${f}_{\text{ctx}}$ will be further processed by FANet. Since those 2 types of features are provided separately, FANet can handle them respectively and balance their relative importance, which will be introduced in the next subsection.

**Table 1:** The feature extraction methods used in this paper

Feature	Abbreviation	Description
One-hot vector	Seq	It composed by 20 types of different amino acids and a 20D one-hot vector is used to encode it
Position-specific scoring matrix	PSSM	It represents the probabilities of 20 amino acids occurring at each position, and the PSI-BLAST algorithm is used to generate it, i.e., we search against the NCBI’s non-redundant sequence database with three iterations and an E-value threshold 0.001
Entropy density	Den	It represents the composition information of the protein sequence and obtained by calculating the information entropy of 20 amino acid residues
Physicochemical properties	PhyChem	It represents the physical and chemical attributes of different amino acid residues and obtained by multivariate statistical analysis of 188 natural amino acid properties
Hydrophilicity and hydrophobicity index	HyIn	A larger hydropathic index means that the residue is more hydrophilic. Conversely, the residues will have higher hydrophobic properties. The hydrophobicity index is the opposite
Pseudo amino acid based on K-nearest neighbors	K-PseAA	It is a new feature combining K-nearest neighbors with the PseAA proposed in this paper. A subsequence is formed by combining the targeted amino acid residue with the residues that are not more than K before and after it. The length of the subsequence is 2K + 1. Then we calculate the PseAA of this subsequence as the K-PseAA feature of the targeted amino acid residue

### The feature aggregation network

The context feature ${f}_{\text{ctx}}$ provided by FFMod is a vector sequence. If the targeted residue feature ${f}_{\text{tr}}$ is directly concatenated with ${f}_{\text{ctx}}$ to form a full representation of the input protein, the dimension of the context feature is overwhelming, preventing the classifier to exploit the target residue feature. The goal of the FANet is to generate an aggregated feature vector ${f}_{\text{agg}}$ from ${f}_{\text{ctx}}$, whose dimension is comparable to the dimension of ${f}_{\text{tr}}$. Correspondingly, FANet contains 2 paths for the targeted residue feature and the context feature respectively, as shown in Fig. 2. The main path performs the feature aggregation for the context feature ${f}_{\text{ctx}}$. The input vector sequence is first processed by a deep convolutional neural network (DCNN) [44] block, consisting of convolution, ReLU and max pooling operations. In this phase, 1D convolution [45] is applied with zero padding so that the output sequence maintains the same length as the input. In this way, local invariant patterns can be captured by this module. Then the output of the DCNN block is further processed by a multi-head self-attention module, which assigns different weights to the features. For each element ${z}_{i}$, a query ${q}_{i}$, a key ${k}_{i}$ and a value ${v}_{i}$ are generated by the weight matrices ${W}_{Q}$, ${W}_{K}$ and ${W}_{V}$ as follows:

1 $$\begin{aligned} & q_{i} = W_{Q} z_{i} ,k_{i} = W_{K} z_{i} ,v_{i} = W_{V} z_{i} , \\ & W_{Q} ,W_{K} , W_{V} \in {\text{R}}^{{d_{m} \times D}} ,q_{i} ,k_{i} ,v_{i} \in {\text{R}}^{{d_{m} }} \\ \end{aligned}$$

where ${d}_{m}$ is the feature dimension of each head, and $D$ refers to the total number of convolutional filters in DCNN block. By matrices, Eq. (1) can be rewritten as:

2 $$\begin{array}{*{20}l} {Q = W_{Q} Z, K = W_{K} Z, V = W_{V} Z,} \hfill \\ {Q, K, V \in {\text{R}}^{{d_{m} \times L}} } \hfill \\ \end{array}$$

To calculate the attention weights, an energy score matrix $E$ is calculated with a Mask operation:

3 $$E = Mask\left( {\frac{{Q \times K^{T} }}{{\sqrt {d_{m} } }}} \right)$$

where the correlation matrix $Q \times {K}^{T}$ is scaled by $\sqrt{{d}_{m}}$ [46]. The Mask operation adds a large penalty to each position in the padding regions, which weakens the attention to those regions. After that, the weights are obtained by a softmax function as follows:

4 $$w_{i,j} = \frac{{exp\left( {e_{i,j} } \right)}}{{\mathop \sum \nolimits_{i = 1}^{L} exp\left( {e_{i,j} } \right)}},\quad 1 \le i, j \le L$$

where $L$ is the sequence length produced by DCNN block.

Then, the feature of this head at position *j* is a weighted summation defined as:

5 $$h_{j} = \sum\nolimits_{i = 1}^{L} {w_{i,j} v_{i} ,\quad 1 \le j \le L}$$

6 $$H_{h} = \left[ {h_{1} , \ldots ,h_{j} , \ldots ,h_{L} } \right]$$

By concatenation, the multi-head features are obtained by:

7 $${\mathbf{A}} = \left[ {a_{1} , \ldots ,a_{j} , \ldots ,a_{L} } \right] = {\text{concat}}\left( {H_{h} } \right), \quad 1 \le h \le d_{H}$$

where $H_{h}$ is the output feature of a head, $d_{H}$ is the number of heads. The obtained sequential features $z_{i}$ and $a_{i}$, $1 \le i \le L$, are concatenated in an elementwise manner as Eq. (8):

8 $${\mathbf{S}} = \left[ {s_{1} , \ldots ,s_{j} , \ldots ,s_{L} } \right], s_{i} = {\text{concat}}\left( {z_{i} , a_{i} } \right),\quad 1 \le i \le L$$

By concatenation, the features from 2 levels of abstraction can be maintained. Next, an average pooling is adopted across all the elements in $\mathbf{S}$, aggregating all features into an information-dense vector as the abstraction of the input sequence.

9 $$f_{{{\text{agg}}}} = {\text{AveragePooling}}\left( {\mathbf{S}} \right)$$

The protein feature ${f}_{\text{prot}}$ is the concatenation of ${f}_{\text{agg}}$ and ${f}_{\text{tr}}$, which passes through another path without any transformation. Following traditional design patterns, the input ${f}_{\text{tr}}$ should be transformed by several fully connected layers. However, ${f}_{\text{tr}}$ will be processed by the fully connected layers in the following PPIPred module. It is not necessary to add extra fully connected layers in this path to save some parameters. Similarly, there is no need to add fully connected layers to adjust the dimension of ${f}_{\text{ctx}}$. Instead, the numbers of convolution kernels and attention heads are carefully controlled so that the dimensions of ${f}_{\text{tr}}$ and ${f}_{\text{agg}}$ are comparable.

### The predictor of protein–protein interaction sites

The PPIPred module consists of 3 fully connected layers (FC Layers) with ReLU activation as shown in Fig. 2. To smooth the training, batch normalization is inserted between adjacent fully connected layers. Similarly, DropOut is applied to enhance the generalization. The prediction is produced by a sigmoid activation**.**

### The stacking of multiple base classifiers

As a matter of fact, model performance heavily relies on features. One can conduct a series of experiments to find the optimal feature combinations and train one PPINet as the predictor. However, the results could be misleading due to overfitting when those experiments are based on limited data. As a better alternative, multiple PPINets are trained and then ensembled via stacking [47] in this paper. The ensembled model, called StackingPPINet, could be more robust thanks to the model diversity. To obtain diverse individual PPINets, each PPINet is trained independently using different data and feature combinations.

Suppose there are $K$ combinations features, each combination corresponding a base classifier which is employed in StackingPPINet. The parameters $K$ is three in this paper. With different feature combinations, multiple PPINets can be trained independently. Then, these diverse models are ensembled via stacking. The final prediction is made by a decision rule in the stacking module as shown in Fig. 2.

### Benchmark datasets

In the process of model hyperparameter adjustment, three benchmark datasets, i.e., Dset_186, Dset_72 [48] and PDBset_164 datasets [31], are fused as a dataset, called Dset_186_72_PDB164 in this paper. To maintain consistency with other model training data, we remove two protein sequences as they do not have the definition of secondary structure of proteins (DSSP) file, same as the datasets in [32]. In the fact, we do not use the DSSP feature. There are 422 protein sequences ranging from 39 to 2000 amino acids in the fused dataset, and 61.85% of them contain less than 200 amino acids. An amino acid is defined as a protein–protein interaction (PPI) site if its absolute solvent accessibility is < 1 Å^2^, otherwise, it is a non-PPI site. There are 13,536 interaction sites and 74,504 non-interaction sites. Table 2 shows the statistics of those datasets. Dset_186_72_PDB164 is divided into a training set, a validation set, and a test set according to the ratio of 3:1:1, respectively. The divided process complies with two principles, they are random selection, and sites of the same sequence are in the same sub-dataset.

**Table 2:** The statistics of all sites in Benchmark datasets

Dataset	Sequences			Interaction sites		Non-interaction sites	All sites
	Number	Average length	Length ≤ 200 (%)	Number	Proportion (%)		
Dset_186	186	195	65.05	5517	15.23	30,702	36,219
Dset_72	72	252	56.94	1923	10.6	16,217	18,140
PDBset_164	164	205	60.37	6096	18.1	27,585	33,681
Dset_186_72_PDB164	422	209	61.85	13,536	15.37	74,504	88,040
Dset_448	448	260	35.94	15,810	13.57	100,690	116,500
The large dataset	9982	426	28.01	427,687	10.05	3,826,511	4,254,198

In the comparison with other methods, we first compare the trained model on Dset_186_72_PDB164 with the performance in [32]. The paper uses the fused dataset for model training. And then we evaluate our proposed method with the performance in [17]. We use a large dataset [49] as this paper to train our model, and then do the same test on Dset_448 [50]. The raw data of Dset_448 was from the BioLip database [51]. The statistics of sites in the two datasets show on Table 2.

It is well acknowledged that similar sequences between training and testing datasets negatively affect the generalization of the evaluated performance of a machine learning model. Dset_186 was built based on a PDB collection [52] to which a six-step filtering process was applied to refine the data, including similarities elimination. Dset_72 was constructed based on the protein–protein benchmark set version 3.0 [53], and any sequences showing ≥ 25% sequence identity over a 90% overlap with any of the sequences in Dset_186, using BLASTClust, were removed. Dset_164 with the same filtering technique as for Dset_186 and Dset_72. The raw data was further processed by removing protein fragments, mapping BioLip sequences to UniProt sequences, and clustering, so no similarities above 25% are shared within Dset_448. The sequences from the large training dataset sharing similarities above 25% were removed, as measured by PSI-CD-hit [54].

### Data balancing strategy

Since the data sets for PPI site prediction problem are usually highly unbalanced, traditional oversampling and subsampling methods do not work well. Here, we first construct a series of subsets, where the samples are relatively balanced. Then, we use subsampling to balance all the subsets, which are used for model training. To do so, we first compute the ratio between PPI sites and non-PPI sites, as shown in Eq. (10):

10 $$M = { }\frac{{N_{r\_n} }}{{N_{r\_p} }}$$

where ${N}_{r\_n}$ and ${N}_{r\_p}$ are non-PPI sites and PPI sites in the dataset. Usually, non-PPI sites are far more than PPI sites. Hence, $M>1$. Then, we divide non-PPI sites into $M$ parts. Each part of the non-PPI sites is combined with all PPI sites to form a subset, where the ratio of non-PPI sites to PPI sites is less than 2. The constructed $M$ subsets are fed to the PPINets for training. During training, each subset is further balanced by subsampling. In this way, when all non-PPI sites are fed to the networks, PPI sites have been learned $M$ times. To some extent, PPI sites are oversampled.

### Implementation details

Our model is implemented by PyTorch (http://pytorch.org/). The loss function for StackingPPINet is mean square error (MSE), while the loss for training the individual PPINet is the cross-entropy loss, defined as follows:

11 $$Loss = - \frac{1}{n}\sum {\left[ {y\log \left( {y_{pred} } \right) + \left( {1 - y} \right)\log \left( {1 - y_{pred} } \right)} \right]}$$

where $n$ is the number of all training samples, $y$ is the label and ${y}_{pred}$ is the model prediction.

The program is written in Python 3.7.4 with PyTorch 1.8.1 + cu101 as the back end. All features are computed from protein sequences only. According to the methods proposed in [38–43], we have implemented feature extraction functions used in the paper in Python, which have been published on GitHub. The parameters of the feature extraction methods are given in Table 3. The structure and parameters of the model are shown in Table 4. The length of the sliding window for context features is discussed in the experimental section, where the window length of 8, 16, 32, and 64 are considered. The threshold is set to 0.5 for the final decision.

**Table 3:** The parameters of the feature extraction method in each FFMod

Component	FFMod	Parameter	Value
Seq	0	Dimension	20
Den	0	Dimension	20
PhyChem	1	Dimension	21
HyIn	1	Dimension	2
PSSM	2	Dimension	20
K-PseAA	2	Max level correlation factor	10
		Dimension	30

**Table 4:** The modules and parameters of the model in the experiment

Component	FFMod	Parameter	Value
Convolutional layers	0,1,2	Kernel size (1-Dimensional)	5,5,5
		Number of Kernels	8,8,8
		Strides	1,1,1
		Activation function	ReLU
Pooling layers	0,1,2	Size (1-Dimensional)	3,3,3
		Strides	1,1,1
Self-attention	0,1,2	Heads	4
		Attention-dimension	16
Fully connected layer	0,1,2	Neurons	1024
		Neurons	256
		Neurons	1
		Activation function	ReLU,ReLU,Sigmoid
		DropOut rate	0.5

We trained our model on the training set with the Adam optimizer [55]. To avoid overfitting, DropOut is applied after the first and the second fully connected layer with the rate of 0.5. The training stops when the average loss of the last 3 epochs continues to increase for 5 epochs or the maximum epochs of 50 is reached. Meanwhile, the independent validation set is also used to tune hyper parameters and perform model selection, such as choosing different ensemble methods and convolutional neural network architectures. Finally, the model is tested on an independent test set. The training and testing are conducted on a workstation with a GTX 1660Ti graphics card and 32 GB RAM. The training parameters are listed in Table 5.

**Table 5:** The training parameters in experiment

Parameter	Value
Optimizer	Adam with default parameters
Learning rate	0.001
Batch size	64
Max epoch	50

## Results

### Evaluation metrics

We assume the PPI sites are the positive samples and the non-PPI sites are the negative samples. To evaluate the performance, we use five evaluation metrics. They are accuracy (ACC), precision (Pre), recall (Rec), F1 scores (F1) and Matthew’s correlation coefficient (MCC). The calculations of these measurements are:

12 $$ACC = \frac{TP + TN}{{TP + TN + FP + FN}}$$

13 $$Pre = \frac{TP}{{TP + FP}}$$

14 $$Rec = \frac{TP}{{TP + FN}}$$

15 $$F1 = \frac{2*Pre*Rec}{{Pre + Rec}}$$

16 $$MCC = \frac{TP*TN - FP*FN}{{\sqrt {\left( {TP + FP} \right)\left( {TP + FN} \right)\left( {TN + FP} \right)\left( {TN + FN} \right)} }}$$

where $\mathrm{TP}$, $\mathrm{TN}, \mathrm{FP}$ and $\mathrm{FN}$ represent true positives, true negatives, false positives, and false negatives, respectively. Area under the ROC curve (AUROC) and area under the precision-recall curve (AUPRC) are also used for evaluations [56].

### Performance comparison of StackingPPINet and other PPI predictors

To evaluate the performance of the proposed method, we have compared it with six state-of-the-art machine-learning-based methods on the Dset_186_72_PDB164. They are PSIVER [48], SPPIDER [30], SPRINGS [31], ISIS [29], RF_PPI [28] and DeepPPISP [32]. PSIVER uses the PSSM and solvent accessibility within a sliding window to represent the feature of the targeted residue site, and it employs a naive Bayes classifier for prediction. RF_PPI uses a variety of feature representations and employs a random forest classifier for PPI sites prediction. The other four model are described in Section Background. Among these methods, ISIS, SPRINGS and DeepPPISP are neural network models, while SPPIDER uses a SVM classifier.

In the experiment, we use the same datasets to train our model as the other six methods. At last, we use the same set as the other six methods for testing. Table 6 shows the predictive performance of different methods. It can be seen from the experiment results that StackingPPINet achieves better performance than the other algorithms in terms of all evaluation metrics except ACC. With respect to Rec, our method obtains the highest value of 0.683, which is 0.106 over the second-best method. For Pre, F1 and MCC, the results of our method also demonstrate significant advantages over those of the completing alternatives. In summary, these results clearly show the superiority of our method in reliably predicting the PPI sites. As the DeepPPISP achieves suboptimal performance on the aggregate metrics, we further compare the AUROC and AUPRC of StackingPPINet with DeepPPISP. The AUROC considers the classification of positive and negative samples at the same time. It can be seen that the performance of StackingPPINet and DeepPPISP is basically the same. AUPRC is better suited to evaluate unbalanced data classification. On this metric, the performance of StackingPPINet is clearly better than that of the DeepPPISP. In addition, DeepPPISP uses the secondary structure information of protein sequences. Compared with DeepPPISP, the features used in our model uses are easier to obtain.

**Table 6:** Predictive performance of different methods on the Dset_186_72_PDB164

Method	ACC	Pre	Rec	F1	MCC	AUROC	AUPRC
PSIVER	0.653	0.253	0.468	0.328	0.138		
SPPIDER	0.622	0.209	0.459	0.287	0.089		
SPRINGS	0.631	0.248	0.598	0.35	0.181		
ISIS	0.694	0.211	0.362	0.267	0.097		
RF_PPI	0.598	0.173	0.512	0.258	0.118		
DeepPPISP	0.655	0.303	0.577	0.397	0.206	0.65	0.68
StackingPPINet	0.597	0.530	0.683	0.582	0.226	0.65	0.77

Table 7 provides the *p* values of the two-tailed t-test for the metrics on the Dset_186_72_PDB164 data set. From this table, it can be seen that StackingPPINet considerably outperforms other methods in terms of Precision, Recall, and F1. For MCC, StackingPPINet outperforms other methods except for SPRINGS and DeepPPISP. SPRINGS achieves a similar MCC as StackingPPINet, while DeepPPISP obtains significantly better MCC than StackingPPINet. In addition, DeepPPISP performs slightly better than StackingPPINet in terms of AUROC and AUPRC, with the *p* values of 0.0479 and 0.0593, respectively.

**Table 7:** The *p* values of the two-tailed *t*-test for the metrics on the Dset_186_72_PDB164

Method	*p* value of Pre	*p* value of Rec	*p* value of F1	*p* value of MCC
PSIVER	< 0.0001	< 0.0001	< 0.0001	0.1158
SPPIDER	< 0.0001	< 0.0001	< 0.0001	0.0008
SPRINGS	< 0.0001	0.0008	< 0.0001	0.3024
ISIS	< 0.0001	< 0.0001	< 0.0001	0.0016
RF_PPI	< 0.0001	< 0.0001	< 0.0001	0.0136
DeepPPISP	< 0.0001	< 0.0001	< 0.0001	0.0228

To further evaluate the performance of our proposed method, we also compared it with nine state-of-the-art machine-learning-based methods on the Dset_448. They are SCRIBER [50], SSWRF [36], SPRINT [57], CRFPPI [35], LORIS [14], SPRINGS [31], PSIVER [48], SPPIDER [30] and DELPHI [17]. They are also sequence-based methods as sequence information is readily available for most proteins. The evaluation of the other programmers comes from [17]. In the experiment, we use the same datasets to train our model as the other nine methods. Table 8 shows the predictive performance of different methods.

**Table 8:** Predictive performance of different methods on the Dset_448

Method	Pre	Rec	F1	MCC	AUROC	AUPRC
SPPIDER	0.194	0.202	0.198	0.071	0.517	0.159
SPRINT	0.183	0.183	0.183	0.057	0.570	0.167
PSIVER	0.191	0.191	0.191	0.066	0.581	0.170
SPRINGS	0.228	0.229	0.229	0.111	0.625	0.201
LORIS	0.263	0.264	0.263	0.151	0.656	0.228
CRFPPI	0.264	0.268	0.266	0.154	0.681	0.238
SSWRF	0.286	0.288	0.287	0.178	0.687	0.256
SCRIBER	0.332	0.334	0.333	0.230	0.715	0.287
DELPHI	0.371	0.371	0.371	0.272	0.737	0.337
StackingPPINet	0.360	0.418	0.387	0.129	0.593	0.406

It can be seen from the experiment results that StackingPPINet achieves the best performance in the most important metrics, such as AUPRC and Rec. It surpasses the second-best programmer by 0.069 and 0.047, respectively. This shows that our algorithm achieves the best results when considering both interaction and non-interaction sites on unbalanced datasets.

## Discussion

We introduce an ensemble framework, StackingPPINet, for PPI sites prediction. To demonstrate its performances, we compare it with twelve other PPI sites prediction methods. Based on the design of StackingPPINet and the results of the experiments, we identified five issues worth further discussion. They are the effect of balancing dataset, the stacking ensemble method and its integrated rules, the effectiveness of hybrid feature, the performance on sequences of different lengths. We discuss these issues as follows.

### The improvement of using multiple balanced datasets

In the experiment, we compare the predictive performance of the classifiers trained by the unbalanced datasets and the balanced datasets under the same model settings, respectively. We construct the balanced datasets as above described. When training with an unbalanced dataset, the training dataset for each epoch is the entire original dataset. The model structure and parameters of the two experiments are the same. The difference between the two is only whether the datasets using in the training process is processed with the balance strategy proposed in the paper. The stacking adopts the logistic regression to integrate the primary results. The parameters of the classifier model are detailed in Table 4. The length of sliding window for the context feature are 16.

**Fig. 3:**
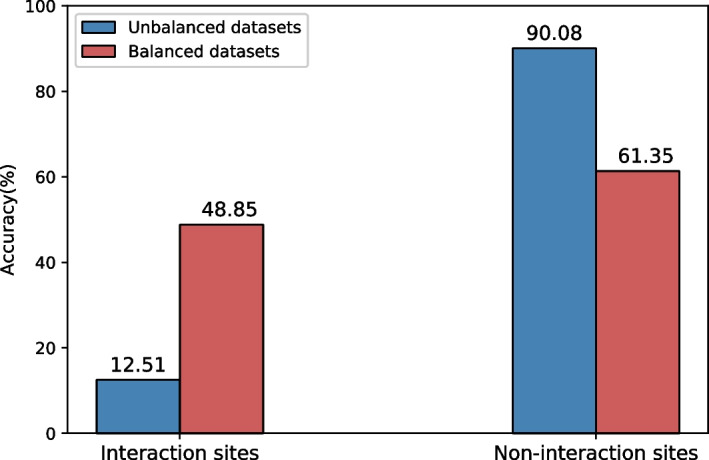
The accuracy of interaction and non-interaction sites obtained by unbalanced and balanced datasets

Table 9 shows the performance of our model using unbalanced and balanced datasets. ACC, Pre, Rec, F1, and MCC obtained with the balanced datasets are 0.549, 0.489, 0.565, 0.512, and 0.105, respectively. The results under all evaluation metrics are improved comparing with the results trained with unbalanced datasets except ACC. Especially in F1 and MCC, which reflect the comprehensive performance, the indicators increase from 0.111 to 0.512, and from 0.025 to 0.105, respectively. Since non-interaction sites are far more abundant than interaction sites, the classifier is inclined to the majority category, which simply achieves high ACC and produce deceptive performance. Figure 3 shows the accuracy of interaction and non-interaction sites obtained by the unbalanced and the balanced datasets, respectively. The precision of the non-PPI sites obtained by the unbalanced datasets is relatively high, while the precision of the PPI sites is extremely low. As a matter of fact, it is more important to correctly classify PPI sites than non-PPI sites in practice. Therefore, we use multiple balanced datasets to improve the precision of the PPI sites.

**Table 9:** Predictive performance of using unbalanced and balanced datasets

Method	ACC	Pre	Rec	F1	MCC	AUROC	AUPRC
Unbalanced datasets	0.795	0.076	0.205	0.111	0.025	0.533	0.189
Balanced datasets	0.549	0.489	0.565	0.512	0.105	0.571	0.554

### The improvement by stacking

In the proposed method, we use a stacking ensemble method to integrate the prediction of primary classifiers. We here focus on whether the PPI sites prediction could indeed benefit from the stacking method. To this end, we keep the other parts of our model unchanged and replace the ensemble method with either a voting or an averaging mechanism for final prediction. We then compare the prediction results obtained by the three models. Among them, stacking adopts logistic regression as the ensemble rule. The parameters of the classifier are detailed in Table 4. The length of sliding window for the context feature are 16.

Table 10 shows the performance of the three ensemble methods for predicting PPI sites. The voting method does not end up with a probabilistic calculation, so its AUROC and AUPRC values are not calculated. Obviously, the stacking achieves the best Rec, MCC, AUROC and AUPRC while the averaging mechanism reaches the optimal values on the other three metrics. Although the voting method does not obtain the best results under any evaluation metrics, it retains a relatively stable performance.

**Table 10:** Predictive performance of using different ensemble methods

Method	ACC	Pre	Rec	F1	MCC	AUROC	AUPRC
Stacking	0.549	0.489	0.565	0.512	0.105	0.571	0.554
Voting	0.531	0.586	0.532	0.557	0.062		
Averaging	0.552	0.616	0.549	0.581	0.104	0.565	0.551

In addition, we compared the predictions of individual frames and their integrated with stacking. Table 11 shows the performance results. With stacking, the AUROC and AUPRC values are increased, and the model has stronger generalization ability.

**Table 11:** Predictive performance of using and not using stacking

Method	ACC	Precision	Recall	F_value	MCC	AUROC	AUPRC
PPINet 0	0.508	0.508	0.710	0.592	0.013	0.509	0.516
PPINet 1	0.527	0.540	0.425	0.475	0.057	0.549	0.539
PPINet 2	0.543	0.544	0.578	0.560	0.087	0.562	0.551
Stacking	0.549	0.489	0.565	0.512	0.105	0.571	0.554

### The effects of different integrated rules in stacking

In the stacking ensemble method, different integration rules could also impact the prediction results of PPI sites. In our experiments, we compare the prediction results obtained by four different stacking rules, i.e., logistic regression, decision tree, random forest, and nearest neighbor. The parameters of the classifiers are listed in Table 4. The length of sliding window for the context feature are 16.

Table 12 shows the performance of four stacking rules. The logistic regression achieves the best results on all indicators except Pre. Notably, on MCC, its performance is significantly better than the other alternatives, indicating its overall superiority. Taken together, the comparison results show that the logistic regression could obtain better performance than the other three integrated rules.

**Table 12:** Predictive performance of using different integrated rules

Method	ACC	Pre	Rec	F1	MCC	AUROC	AUPRC
Logistic regression	0.549	0.489	0.565	0.512	0.105	0.571	0.554
Decision tree	0.513	0.476	0.518	0.496	0.027	0.492	0.518
Random forest	0.518	0.482	0.524	0.501	0.038	0.485	0.490
Nearest neighbor	0.516	0.497	0.521	0.507	0.033	0.496	0.527

### The effectiveness of hybrid feature

In this subsection, we exhibit the effectiveness of feature combination. We first compare the results using feature combination with the results using individual feature. Then we investigate the predictive performance under different length of sliding window for the context feature. The parameters of the models are listed in Table 4.

**Fig. 4:**
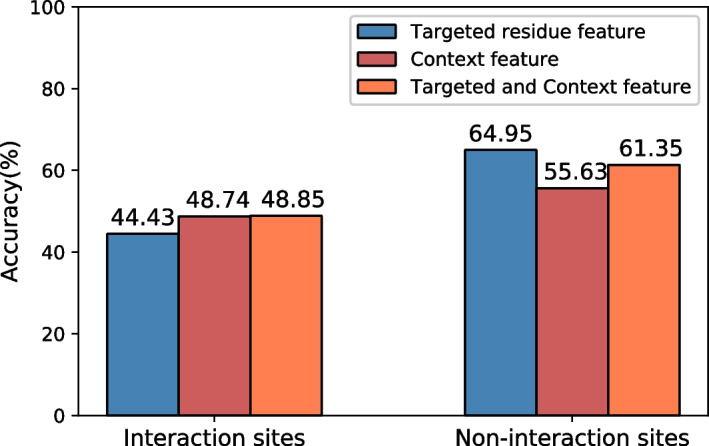
The accuracy of interaction and non-interaction sites with different features

We compare the predictive performance obtained by different features in experiments. Except the processing module of the context feature and the targeted residue feature, the rest parts are the same. The length of sliding window for the context feature are 16. The detailed results are shown in Table 13. After adding the targeted residue feature, the predictive performance is improved under all evaluation metrics. Specifically, two comprehensive indicators F1 and MCC, increase from 0.499 to 0.521, and 0.046 to 0.105, respectively. This indicates that the targeted residue feature is important to the decision making. Figure 4 shows the improvement of the feature connection for PPI and non-PPI sites. We can see that the addition of the targeted residue feature really improves the accuracy of PPI sites and non-PPI sites.

**Table 13:** The effectiveness of feature combination

Method	ACC	Pre	Rec	F1	MCC	AUROC	AUPRC
Targeted and context feature	0.549	0.5	0.567	0.521	0.105	0.574	0.575
Targeted residue feature	0.544	0.444	0.568	0.488	0.099	0.567	0.568
Context feature	0.52	0.487	0.532	0.499	0.046	0.54	0.538

### The effect of sliding window length

The length of sliding window for the context feature is the amino acid range that characterizes the biological properties of the targeted site. In the experiments, we compare the effects of different lengths on the model performance by keeping other model hyper parameters unchanged while varying the lengths of sliding window. It can be seen from the results that the lengths are not as large as possible. If the value is too small, the amino acid residues in the range cannot fully reflect the biological properties. If the value is too large, some amino acid residues in the range may not be related to the interaction of the targeted site. We find that when the length of sliding window for the context feature is 32, our model could reach the best performance. Table 14 shows the effects of different sliding window lengths.

**Table 14:** The effects of different sliding window lengths

Length	ACC	Pre	Rec	F1	MCC	AUROC	AUPRC
8	0.527	0.461	0.541	0.491	0.060	0.545	0.542
16	0.549	0.489	0.565	0.512	0.105	0.571	0.554
32	0.549	0.500	0.567	0.521	0.105	0.574	0.575
64	0.503	0.471	0.500	0.479	0.005	0.502	0.503

### The performance on sequences in different lengths

**Fig. 5:**
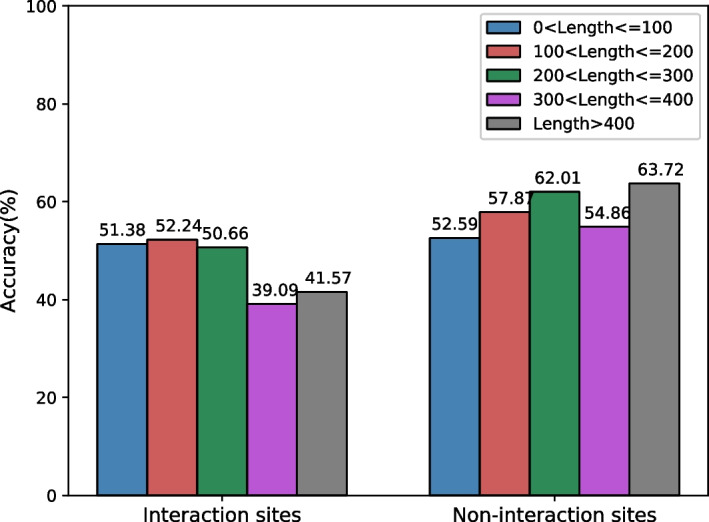
The accuracy of interaction and non-interaction sites on two categories

In the experiment, we divide the test set into several subsets and show how performance varies according to the sequence length. As shown in Table 15, the best performance is achieved when the sequence length falls between 100 and 300. When the sequence is too short, the model obtains limited information from the input; when the sequence is too long, the model can be misled by redundant or irrelevant information. Either of those cases may harm the performance, especially for MCC, AUROC, and AUPRC. We also illustrate the accuracy of interaction and non-interaction sites for each subset in Fig. 5. It shows that the accuracy gap rises considerably when the sequence is longer than 200. When the sequence is longer than 400, the accuracy for interaction sites is 22.15% lower than the non-interaction sites. The possible reason could be the data imbalance. There are always enough non-interaction samples (negative samples) for training, while long interaction samples (positive samples) are relatively limited and more difficult to learn. Thus, for long sequences, our model can get high accuracy for non-interaction samples but considerably lower accuracy for interaction samples.

**Table 15:** Predictive performance on sequences in different lengths

Length	ACC	Pre	Rec	F1	MCC	AUROC	AUPRC
< 100	0.525	0.514	0.777	0.608	0.035	0.508	0.773
100–200	0.548	0.522	0.641	0.562	0.098	0.581	0.643
200–300	0.542	0.507	0.577	0.501	0.121	0.586	0.554
300–400	0.566	0.391	0.439	0.406	0.063	0.549	0.423
> 400	0.562	0.416	0.4	0.395	0.055	0.532	0.397

### The effect of multi-head attention

In the PPINet, context features are processed by a CNN block and the multi-head self-attention. Convolutional layers extract features locally, while self-attention aggregates all the sequence information globally. By combining them, it is expected to obtain the global representation of a sequence more efficiently. If the self-attention is removed, one has to add more convolutional layers to extend the reception field to cover the whole sequence. To show the effectiveness of multi-head self-attention, in this experiment, the performance of the model with multi-head attention, the model with single-head attention, and the one without attention are compared. As shown in Table 16, the Base-Model is the one introduced in Table 4 with 4-head self-attention. The SH-Model has the same setup as the Base-Model except that the number of attention heads is only 1. The last NA-Model is constructed by removing the self-attention from the Base-Model and only contains convolutional and fully connected layers. The results show that SH-Model and NA-Model achieve similar ACC, Rec, MCC, AUROC, and AUPRC. SH-Model gains higher Pre and F1. Base-Model outperforms the other two models in all the metrics, indicating the effectiveness of the multi-head attention in global feature aggregation.

**Table 16:** Performance comparison of the models with different self-attention setup

Model	ACC	Pre	Rec	F1	MCC	AUROC	AUPRC
Base-model (4-head attention)	0.549	0.489	0.565	0.512	0.105	0.571	0.554
SH-model (Single-head attention)	0.528	0.454	0.540	0.491	0.056	0.539	0.528
NA-model (No attention)	0.522	0.317	0.549	0.375	0.051	0.540	0.543

## Discussion

In the above experiments, only sequence based features are exploited in the proposed model for the sake of fair comparison with considered baseline methods. From the methodology of model ensembling, it can be noticed that the improvement by the proposed ensembling strategy is restricted by the low diversity of based classifiers. To break through such limitation, one feasible way is to introduce multiple types of data, e.g. protein structure features, protein domain features, to train base models. On the one hand, multiple types of information help to construct a full description of a protein; on the other hand, diverse data types require different types of models to process, enhancing the model diversity. Both can bring extra performance gain for model ensembling. The cost of such improvement is the data collection. For a protein, one has to collect multiple types of data to get the prediction, which is not convenient during inference phase. One possible way to further overcome this drawback is to utilize protein language models trained on large sequence data sets. Recent research has reported that accurate protein structure prediction can be achieved by learning from Multiple Sequence Alignment (MSA) data [58, 59] or even pure sequence data [60]. Such models can be used as feature extractors which indirectly introduces protein structure information to base PPINet. However, this method has not been extensively studied yet. We decide to leave it to the further work.

## Conclusions

In this work, we propose a novel sequence-based method for PPI sites prediction from the motivation of extracting more valuable features. Specifically, we extract the single feature of the targeted amino acid residue and the context feature of its neighbors with different combinations of features to compose the hybrid feature. A deep learning framework combined with convolutional neural networks and multi-head self-attention is employed to process the context feature to control these dimensions. In addition, we present a strategy to balance the interaction sites and non-interaction sites so that the model can ultimately learn the original data distribution. This paper compares the proposed method with the prediction algorithms of twelve existing protein–protein interaction sites. The results show that our method performs well in various indicators, especially on the precision of interaction sites. Though the proposed method is demonstrated to have advantages over other competing methods, it also has some limitations. The first is that the model architecture and the features can be extended. The second is that the optimal parameters of the model are obtained through grid search, which is computationally intensive. Future challenges include exploring more efficient feature expression methods and designing more adaptive network architectures.

## Data Availability

The datasets supporting the conclusions of this article are available in the Github repository, https://github.com/CandiceCong/StackingPPINet.

## Abbreviations

PPIs: Protein–protein interactions
NNs: Neural networks
SVMs: Support vector machines
RF: Random forests
FFMod: Feature forming module
FANet: Feature aggregation network
PSSM: Position-specific scoring matrix
Den: Entropy density
PhyChem: Physicochemical properties
HyIn: Hydrophilicity and hydrophobicity index
K-PseAA: The pseudo amino acid based on K-nearest neighbors
FC Layers: Fully connected layers
DCNN: Deep convolutional neural network
AUROC: Area under the ROC curve
AUPRC: Area under the precision-recall curve
